# Expression of Caseicin from *Lacticaseibacillus casei* and *Lacticaseibacillus zeae* Provides Insight into Antilisterial Class IIa Bacteriocins

**DOI:** 10.1007/s12602-024-10341-0

**Published:** 2024-08-13

**Authors:** Francesco Salini, Ross Vermeulen, Anton du Preez van Staden, Giuseppe Comi, Lucilla Iacumin, Leon M. T. Dicks

**Affiliations:** 1 Department of Agriculture, Food, Environmental and Animal Science, University of Udine, Via Sondrio 2/A, 33100 Udine, Italy; 2 Department of Microbiology, Stellenbosch University, Private Bag X1, Matieland, Stellenbosch, 7602 South Africa

**Keywords:** Bacteriocin class IIa, NisP, GFP, Fusion protein, Heterologous expression, Caseicin, *Lacticaseibacillus casei*

## Abstract

In this study, an in silico screening approach was employed to mine potential bacteriocin clusters in genome-sequenced isolates of *Lacticaseibacillus zeae* UD 2202 and *Lacticaseibacillus casei* UD 1001. Two putative undescribed bacteriocin gene clusters (*Cas1* and *Cas2*) closely related to genes encoding class IIa bacteriocins were identified. No bacteriocin activity was recorded when cell-free supernatants of strains UD 2202 and UD 1001 were tested against *Listeria monocytogenes.* Genes encoding caseicin A1 (*casA1*) and caseicin A2 (*casA2)* were heterologously expressed in *Escherichia coli* BL21 (DE3) using the nisin leader peptide cloned in-frame to the C-terminal of the green fluorescent gene (*mgfp5*). Nisin protease (NisP) was used to cleave caseicin A1 (casA1) and caseicin A2 (casA2) from GFP-Nisin leader fusion proteins. Both heterologously expressed peptides (casA1 and casA2) inhibited the growth of *L. monocytogenes*, suggesting that *casA1* and *casA2* are either silent in the wild-type strains or are not secreted in an active form. The minimum inhibitory concentration (MIC) of casA1 and casA2, determined using HPLC-purified peptides, ranged from < 0.2 µg/mL to 12.5 µg/mL when tested against *Listeria ivanovii*, *Listeria monocytogenes*, and *Listeria innocua*, respectively. A higher MIC value (25 µg/mL) was recorded for casA1 and casA2 when *Enterococcus faecium* HKLHS was used as the target. The molecular weight of heterologously expressed casA1 and casA2 is 5.1 and 5.2 kDa, respectively, as determined with tricine-SDS-PAGE. Further research is required to determine if genes within *Cas1* and *Cas2* render immunity to other class IIa bacteriocins.

**Supplementary Information:**

The online version contains supplementary material available at 10.1007/s12602-024-10341-0.

## Introduction

Lactic acid bacteria (LAB) are prolific bacteriocin producers, which allow them to dominate in fermentative environments by targeting similar or closely related species [1], and they are considered safe to use as food preservatives [2]. Bacteriocins currently used as a food preservative are the lantibiotic nisin (E234) and pediocin PA-1/AcH, which is marketed as Nisaplin and Alta™ 2341, respectively [3, 4]. Pediocin PA-1 (AcH) and all bacteriocins belonging to class IIa are active against *Listeria* spp. To date, more than fifty class IIa bacteriocins have been described for the genera *Lactobacillus*, *Enterococcus*, *Pediococcus*, *Carnobacterium*, *Leuconostoc*, *Streptococcus*, *Weissella* [5–7], and non-LAB such as *Bifidobacterium bifidum* [7], *Bifidobacterium infantis* [8], *Bacillus coagulans* [7], and *Listeria innocua* [9]. Despite high similarities in conservative motifs, variations in overall amino acid sequences lead to different inhibitory spectra [10]. All class IIa bacteriocins are not post-translationally modified and have a highly conserved “YGNGV” domain, known as a pediocin box, and “CXXXXCXV” sequence motifs in the N-terminal [11]. Some class IIa bacteriocins with two disulfide bonds require an operon-encoded accessory protein to guide bond formation [12]. The transport of prepeptides across the cell membrane is guided by an N-terminal leader sequence and dedicated ABC transporters.

The regulatory mechanisms involved in the expression of class IIa bacteriocins reveal a general conserved gene arrangement. However, some unusual organizations of biosynthetic gene clusters were also characterized and generally correlated to different transcription regulation systems [10, 13]. Co-culture-based regulation, auto-inducing peptide, acetate, temperature, and divalent cation regulation are independent quorum sensing (QS) triggers, including for bacteriocin production [14–19].

In recent decades, in silico screening, searching for potential new bacteriocins within bacterial genomes has become the major method of screening and predicting protein sequences of interest without depending solely on experimental approaches [20–22]. However, the presence of bacteriocin genes does not always translate into biological antimicrobial activity. Bacteriocin production by wild-type strains is often limited due to difficulties experienced with cultivation and insufficient production of biologically active peptides [23]. The heterologous expression of genes encoding bacteriocins is an attractive and versatile option, especially with the increase in information generated by data mining of genome sequences [24]. In silico screening of sequences and heterologous expression systems may provide more information about the production and regulation of bacteriocins, and the characteristics required from these peptides to serve as food preservatives or control bacterial infections [25, 26].

Here we describe two cryptic class IIa bacteriocin operons, *Cas1* and *Cas2,* located on the genomes of *Lacticaseibacillus zeae* UD 2202 and *Lacticaseibacillus casei* UD 1001, respectively. As observed for pediocin PA-1, sakacin G and plantaricin 423, operons *Cas1* and *Cas2* do not contain classical regulatory genes. In this study, the putative bacteriocin genes *casA1* and *casA2*, encoding bacteriocins caseicin A1 (casA1), and caseicin A2 (casA2) were heterologously expressed in *E. coli* BL21*.*

## Methods

### Bacterial Strains and Culture Conditions

Bacterial growth media were from Biolab (Biolab, Merck, South Africa). *Lactococcus lactis* QU2 (nisin producer), *Lact. zeae* UD 2202 and *Lact. casei* UD 1001 were cultured in De Man, Rogosa and Sharpe (MRS) broth. *Escherichia coli* BL21 (DE3) was cultured in Luria Bertani (LB) broth, supplemented with 1.2% (w/v) agar, and recombinant strains of *E. coli* BL21 (DE3) were cultured in Terrific broth (TB), supplemented with 50 µg/mL Kanamycin (Kan). The test strains *Listeria ivanovi* n29, *Listeria monocytogenes* ATCC 7646, *L. monocytogenes* RTE, *L. monocytogenes* EDG-e, *L. innocua* ATCC 33090, *Enterococcus fecium* HKLHS, *Enterococcus faecalis* S1, and *Clostridiodes difficile* ATCC 70057 were cultured on Brain Heart Infusion (BHI) and supplemented with 1.0% (w/v) agar to prepare solid media. All strains were incubated at 37 °C for 24 h.

### In SilicoGenome Mining and Sequence Analyses

The genome sequences of *Lact. zeae* UD 2202 (GenBank GCA_028878215.1) and *Lact. casei* UD 1001 (GenBank GCA_028878205.1), previously assembled with the pipeline WGA-LP available on GitHub (https://github.com/redsnic/WGA-LP) [27, 28], were mined for putative bacteriocin genes using the Bagel4 software [29]. The Blastx and Blastn command line [30] was used to assess the novelty of the identified peptide sequences and operons listed in the National Center for Biotechnology (NCBI, Bethesda, MD, USA; https://www.ncbi.nlm.nih.gov/ accessed on June 2022). The CLC Main Workbench (CLC bio, Aarhus, Denmark) software was used for the annotation of genetic sequences and map constructions. Characterized Class IIa bacteriocin sequences were acquired from Bactibase [31] and LABioicin [32] repositories. Muscle WS command line [33] was used to perform sequence alignments and visualized by the Tree Of Life (iTOL) v4 online tool [34]. Protein 3D prediction was obtained with ColabFold open-source software available at https://github.com/sokrypton/ColabFold [35].

### Molecular Cloning

DNA was isolated from pure cultures of *Lactococcus lactis* QU2, *Lact. zeae* UD 2202 and *Lact. casei* UD 1001 using the ZR Fungal/Bacterial DNA MiniPrep kit (Zymo Research Corporation, Irvine, CA, USA) according to the manufacturer's instructions. Oligonucleotides were designed using the CLC Main Workbench program (CLC bio, Aarhus, Denmark). DNA concentrations were determined using the BioDrop µLite + (BioDrop Ltd, Cambridge, UK). Amplification via polymerase chain reaction (PCR) was performed according to the Q5 polymerase instruction manual (NEB) in a GeneAmp Thermocycler, model 9700 (ABI, Foster City, CA). T4 DNA ligase and restriction enzymes were acquired from NEB and used according to the manufacturer's instructions.

Plasmid DNA extractions were performed using the PureYield™ Plasmid Miniprep System (Promega, Madison, WI, USA). Gel electrophoresis was at 100 V, using an EphortecTM 3000 V power pack (Triad Scientific, Manasquan USA) with TBE (5:1) as electrophoresis buffer. Gene sequencing was performed by the Central Analytical Facility (CAF), Stellenbosch University (Stellenbosch, South Africa).

### Construction of GFP-Caseicin Expression Systems

Fusion PCR techniques were used to add the nisin leader-amplified nucleotidic sequence from *L. lactis* genomic DNA to *casA1* and *casA2* amplicons encoding mature casA1 and casA2. The putative class IIa bacteriocin genes were cloned into the previously constructed pRSF-GFP vector [36]. In brief, this plasmid contains the N-terminal of the GFP gene, *mgfp5*, fused to a hexa-histidine tag downstream of the T7 promoter. The C-terminal of the GFP gene was modified to include the WELQut cleavage sequence followed by various restriction sites for the expression of GFP-fused genes that can be liberated using the WELQut protease. Plasmid pRSF-GFP was used as a template and linearized with the *Pst*I/*Hind*III restriction site.

Due to their 5’ and 3’ similarity, *casA1* and *casA2* were amplified by PCR from the genomes of *Lact. zeae* UD 2202 and *Lact*. *casei* UD 1001, respectively, using the single primer set FWNispL_casA1/2 Hind_REV_ casA1/2. This primer set installed the flanking *Hin*dIII restriction sites at the 3’ end of *casA1* and *casA2* during amplification. DNA amplification was conducted for 30 cycles, with initial denaturation at 95 °C for 1.30 min, primer annealing for 30 s at 56 °C, and primer extension for 1 min at 72 °C. The truncate *NisA* gene was amplified by PCR from the genomes of *L. lactis* QU2 with primer set GFPNisLeader_PstIF Rev_NisLeaderOri_casA1/2. The amplicon obtained from the truncated *NisA* gene was added in the 5’ *Pst*I restriction site extension. Fusion PCR reactions were carried out in a volume of 50 µL with the same final concentration (20 ng/µL) of amplicons using GFPNisLeader PstIF/Hind_REV_casA1/2 following the same PCR protocol except for the primer annealing step, which was 45 s at 52 °C.

**Fig. 1:**
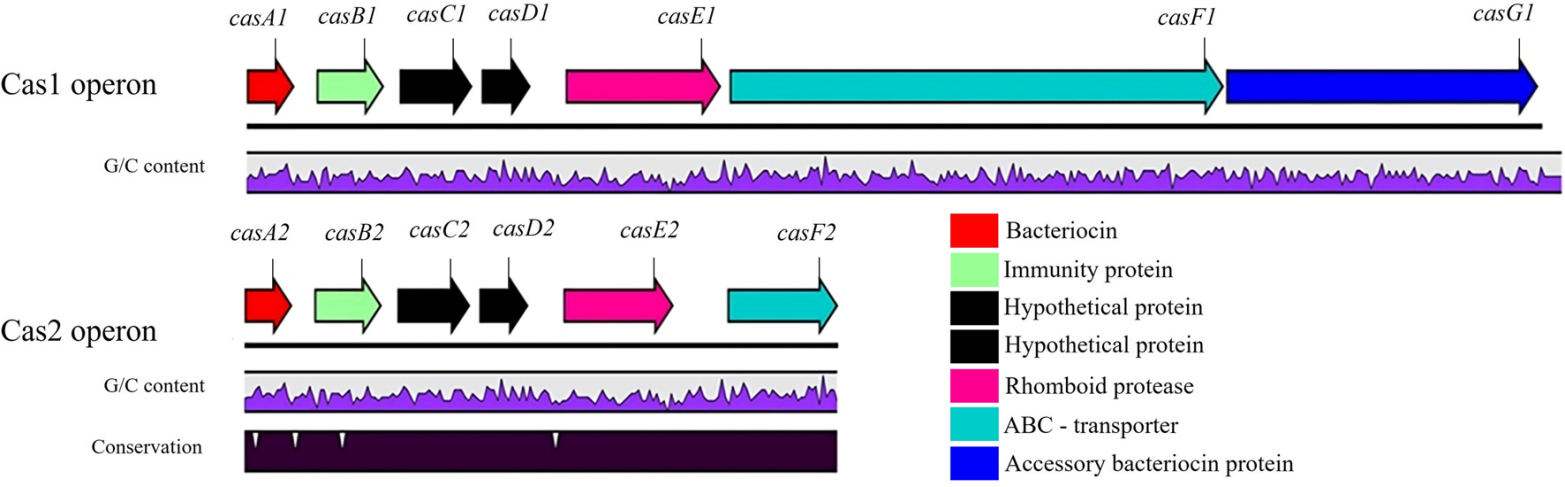
Alignment of genes in the Cas1 and Cas2 operons from the genomes of *Lact. zeae* UD 2202 (GenBank GCA_028878215.1) and *Lact. casei* UD 1001 (GenBank GCA_028878205.1). Both operons contained genes encoding a structural peptide, immunity protein, rhomboid protease, and two hypothetical proteins. The Cas1 operon contains a longer ABC transporter and accessory bacteriocin protein. Neither operon contained genes for classical transcriptional regulatory proteins

The fused genes encoding nisin N-terminal leader and the mature peptides casA1 and casA2 were digested with the *PstI*/*Hin*dIII and ligated using T4 between the WELQut site on a *Pst*I/*Hin*dIII fragment in the linearized pRSF-GFP construct. The pRSF-GFP-NislcasA1 and pRSF-GFP-NislcasA2 constructs allowed for the expression of the GFP-NislCasA1 and GFP-NislCasA2 fusion proteins. Plasmid DNA isolated from transformants was sequenced and transformed into chemically competent *E. coli* BL21 cells. The cells were plated onto BHI agar supplemented with kanamycin (50 µg/mL) and incubated overnight at 37 °C. Single colonies were isolated and used in subsequent expression experiments. Plasmid maps of pRSF-GFP-NislcasA1 and pRSF-GFP-NislcasA2 and 3D visual representations of GFP-CasA1 and GFP-CasA2 fusion proteins are shown in Fig. 1S.

### Construction of the mCherry-NisP Expression System

**Fig. 2:**
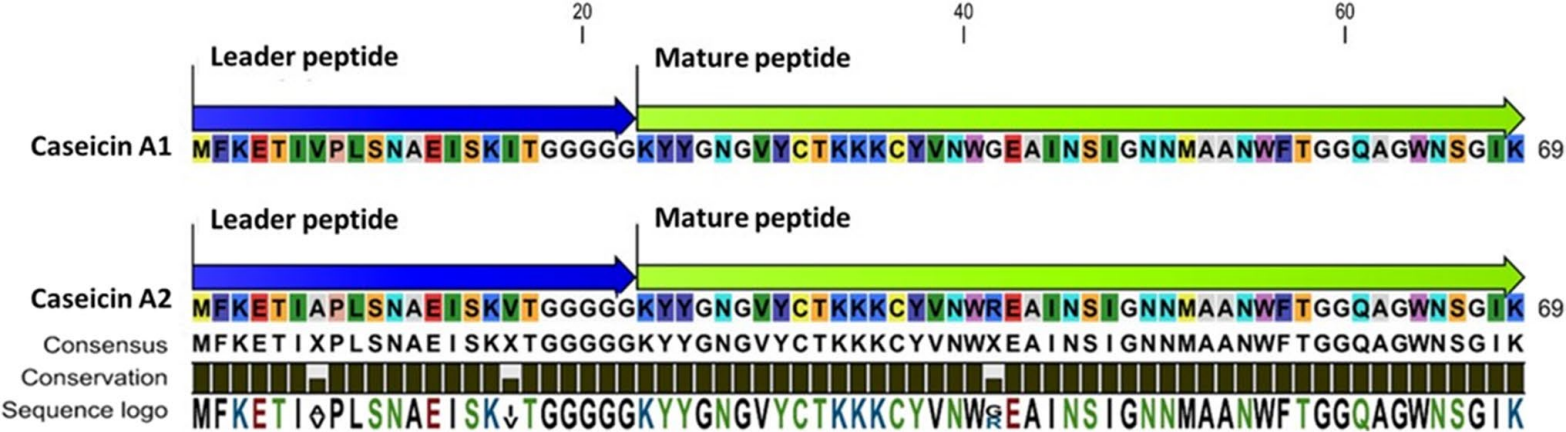
Alignment of amino acids in casA1 and casA2. Substitutions in positions 7 (V or A), 17 (I or V), and 42 (G or R) define the difference in the two peptide variants

The NisP protease expression system (plasmid pRSF-hNisP) was previously constructed to produce soluble NisP (no membrane-spanning domain) with an 8 × C-terminal His tag as reported by Van Staden et al. (2019) [37], but it was noticed that after purification NisP protease tended to precipitation during storage. In unpublished work, the mCherry gene was cloned into the previously constructed pRSF-hNisP plasmid on a double *Hin*dIII amplicon. Briefly, the mCherry gene was amplified using primer set 5'-Fwd_mCherryNisP_Hind-3', 5'-Rev_mCherryNisP_Hind-3'. The amplification of the mCherry gene was conducted for 30 cycles, with initial denaturation at 95 °C for 1.30 min, primer annealing for 30 s at 61 °C, and primer extension for 1 min at 72 °C. To incorporate the mCherry fluorescence gene flanked with *Hin*dIII/*Hin*dIII restriction sites into pRSF-hNisP construct, both were previously digested with *Hin*dIII restriction enzymes. This cloning resulted in the construction of the pRSF-NisP-mCherry8xHis expression plasmid. After molecular cloning and transformation, only red fluorescent transformants were selected for DNA sequencing and subsequent NisP-mCherry fusion protein expression. The map of pRSF-NisP-mCherry8xHis and 3D visual representations of NisP-mCherry fusion proteins are shown in Fig. 2S.

### Expression of Fusion Proteins in *E. coli* BL21

The expression of the fusion proteins was performed as described by [34]. Briefly, *E. coli* BL21 transformants containing the plasmids pRSF-GFP-NislcasA1, pRSF-GFP-NislcasA2, and pRSF-Nisp-mCherry8xHis were respectively inoculated in 5 mL of BHI broth containing 50 µg/mL kanamycin and incubated overnight at 37 °C with constant shaking at 120 rpm.

Overnight cultures were used to inoculate 500 mL (1.0%, v/v) TB, supplemented with 50 µg/mL Kan, and incubated at 37 °C as mentioned elsewhere. At an O.D_600_ of 0.6, protein expression was induced with the addition of 0.1 mM IPTG, and the cultures were incubated at 18 °C for 48 h at 160 rpm on an orbital shaker.

### Purification of Fusion Proteins from *E. coli* BL21

After expression, the *E. coli* cells were harvested (8 000 × g, 20 min, 4 °C) and resuspended in 15 mL/g of SB buffer (Tris 50 mM, NaCl 500 mM, pH 8.0). Cell resuspensions were frozen overnight at − 20 °C. Cell resuspensions were then thawed and lysed with 1 mg/mL lysozyme and incubated at 8 °C for 45 min while stirring. The lysed cells were subjected to sonication (50% amplitude, 2-s pulse, 2-s pause, 6 min) using the Omni Ruptor 400 (Ultrasound Homogenizer, Omni International Inc., Kennesaw, GA). RNaseI and DNaseI (BioLabs, New England) were added to a final concentration of 10 µg/mL and 5 µg/mL, respectively, and then incubated at room temperature for 15 min. The cell lysate was centrifuged for 90 min (20,000 × g, 4 °C), and imidazole was added to the cell-free supernatant to a final concentration of 10 mM. Fusion proteins were purified with immobilized metal affinity chromatography (IMAC) using the super-flow resin Ni–NTA (Qiagen, Germany) equilibrated in SB_10_ buffer (SB buffer containing 10 mM imidazole). Cell-free supernatant containing the protein of interest was loaded directly into the pre-equilibrated Ni–NTA resin. The ÄKTA purifier (Amersham, Biosciences) was used for IMAC purification according to the following program: 5 column volumes (CVs) SB_10_ (2% B: SB_500_), washed with 10 CVs of SB_20_ (4% B: SB_500_ buffer), and eluted with approximately 40 mL of SB_500_ (100%) buffer. Buffer exchange was performed by anion exchange chromatography using the ÄKTA purifier. Each IMAC elution was diluted 35X in AB buffer (50 mM Tris pH 8.3) and loaded onto the DEAE Sepharose fast-flow resin. Elution was performed at 20% AB_1000_ buffer (50 mM Tris 1 M NaCl pH 7.5) on the ÄKTA purifier. Proteins were detected at 280 nm and fractions were collected.

### Bacteriocin Liberation

The NisP-mCherry fusion protein was used for the liberation of CasA1 and CasA2 proteins from their GFP fusion partners. NisP-mCherry was added to GFP-CasA1 and GFP-CasA2 fusion proteins at volume ratio of 1:9, 1:4, 3:7, 4:6, and 1:1 and incubated 4, 16, 30, and 37 °C for 16 h. Cleavage performance was then assessed by spot testing by adding the mixtures in a well diffusion assay in Brain Heart Infusion (BHI) soft agar (0.8%, w/v) seeded with overnight cultures of *L. monocytogenes* EGD-e.

### SDS-PAGE Analysis

Bacteriocins were separated using Tricine-SDS-PAGE electrophoresis (4% stacking gel and 12% running gel). Ten microliters of each sample were loaded into wells, and gels were run in duplicate. One gel was resolved with blue Coomassie, according to Schägger (2006) [38], and the other gel was overlayed with *L. monocytogenes* suspended in BHI soft agar [39].

### Peptide Isolation

Peptides casA1 and casA2 were liberated from GFP using NisP under optimal cleavage conditions. Acetonitrile (75%, v/v) was added, and the suspension was centrifuged at 5000 × g for 5 min to remove GFP and NisP-mCherry. The supernatant was removed and lyophilized. The lyophilized casA1 and casA2 were resuspended in ultrapure water and purified with HPLC-C18 (Poroshell 120 EC-C18 HPLC column 120 Å, 4 µm, 4.6 mm × 150 mm, Agilent) using a linear gradient from 10% A (MilliQ 0.1% TFA) to 60% B (Acetonitrile 0.1% TFA) over 35 min at 1.3 mL/min. Separation was performed on an Agilent 1260 Infinity II LC system. Peaks were collected and spot-tested for antibacterial activity as described elsewhere. Active peaks were collected, pooled, re-lyophilized, and analytically weighed using a XP26 (Mettler-Toledo, USA) balance.

### Antibacterial Spectrum and Minimum Inhibitory Concentrations

Lyophilized casA1 and casA2 were suspended in sterile MilliQ to 50.0, 25.0, 12.5, 6.3, 3.1, 1.6, 0.8, 0.4, and 0.2 µg/mL, respectively. To evaluate the minimum inhibitory concentration (MIC) of casA1 and casA2, a well-agar diffusion test was used. Overnight cultures of *L. ivanovii* n29, *L. monocytogenes* ATCC 7646, *L. monocytogenes* RTE, *L. monocytogenes* EDG-e, *L. innocua* ATCC 33090, *Ent. faecium* HKLHS, *Ent. faecalis* S1, *Clos. difficile* ATCC 70057, *Lact. zeae* UD 2202, and *Lact. casei* UD 1001 were inoculated into 45 mL BHI, DRCM, and MRS to yield a final cell concentration of approximately 1 × 10^7^ cfu/mL. The media were solidified by adding 0.8% (w/v) agar. Wells were made using a sterilized 96-well PCR plate placed into melted inoculated media. Fifty microliters of casA1 and casA2 were spotted against each of the target strains. The MIC was defined as the lowest concentration that produced a clear inhibition zone of ≥ 2 mm after 24 h of incubation at 37 °C. The inhibition halos were highlighted by adding 10 µL resazurin (0.015%, w/v) to each of the wells.

### Scanning Electron Microscopy (SEM)

A single colony of *L. monocytogenes* EGD-e was inoculated in 10 mL sterile BHI and incubated at 37 °C with shaking (120 rpm). After 24 h of incubation, aliquots of 50 µL were added to 50 µL sterile BHI (1:1). Coverslips were exposed to UV light for 30 min and used as solid support. A final volume of 100 µL diluted *L. monocytogenes* EGD-e (approximately 10^7^ cfu/mL) was added to the coverslip top surface and placed in a sterile petri dish (35 × 15 mm). A total of 100 µL of cleaved peptide in 50 mM Tris, 200 mM NaCl buffer were added to the coverslip and incubated at 26 °C overnight. Cells were then fixed with 4% paraformaldehyde (PFA) in PBS (pH 7.2) for 16 h at 4 °C, stained with 2% OsO_4_ for 30 min, washed 3 × with dH_2_0, dehydrated with increments of ethanol (20, 50, 70, 90, 100 v/v) for 5 min, and then sputter-coated with 50 nm gold/palladium. SEM was conducted using a ThermoFisher Apreo FESEM at a beam strength of 2 kV and a current of 20 nA.

## Results

### In SilicoAnalyses

Two putative class IIa bacteriocin operons, *Cas1* and *Cas2*, were identified in *Lact. zeae* UD 2202 and *Lact. casei* UD 1001, respectively (Fig. 1).

The putative genes encoding the two bacteriocins (*casA1* and *casA2*) are each 210 bp, encoding peptides of 69 amino acids. The leader peptides of both bacteriocins have five glycine residues, which differs from leader peptides with two glycine motifs described for other class IIa and IIb bacteriocins. Caseicin A1 (casA1) and caseicin A2 (casA2) core peptides consist of 46 amino acids, including the N-terminal consensus sequence (YGNGV) typical of the pediocin-like bacteriocins characterized by a cationic less-conserved C-terminal region [39]. Moreover, a single disulfide bridge is present in both core peptides that includes four amino acid residues designed between the 2 Cys residues (C_9_TKKKC_14_). The amino acid sequences of casA1 and casA2 are almost identical and only differ at position 7 by having either valine (V) or alanine (A), position 17 by having either isoleucine (I) or V, and by one amino acid within the core peptide sequence at position 42 where glycine (G) in casA1 is substituted for the positively charged arginine (R) residue in casA2 (Fig. 2).

**Fig. 3:**
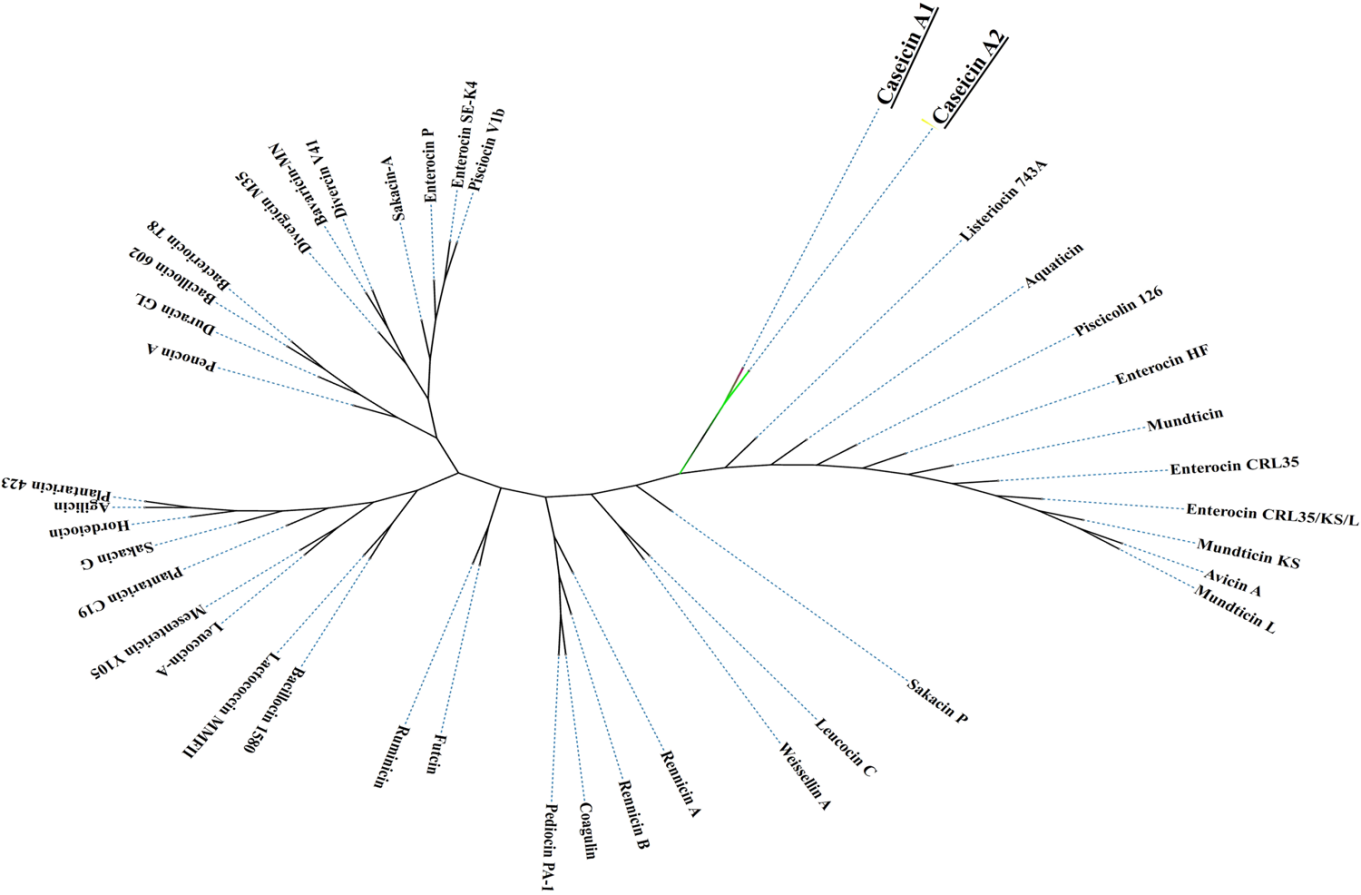
Cladogram alignment of core amino acid sequences from class IIa bacteriocins, including casA1 and casA2

Based on the sequence data presented here, casA1 and casA2 are considered novel and natural co-evolution variants. Multiple sequence alignment (MSA) indicates that casA1 and casA2 do not share the same amino acid pattern with other class IIa bacteriocins (Fig. 3). Mature class IIa listeriocin 743A and sakacin P had the highest amino acid similarity to casA1 and casA2. Additional MSA results are presented in Fig. 3S.

### Expression Plasmid System and Detection of Putative Bacteriocins

**Fig. 4:**
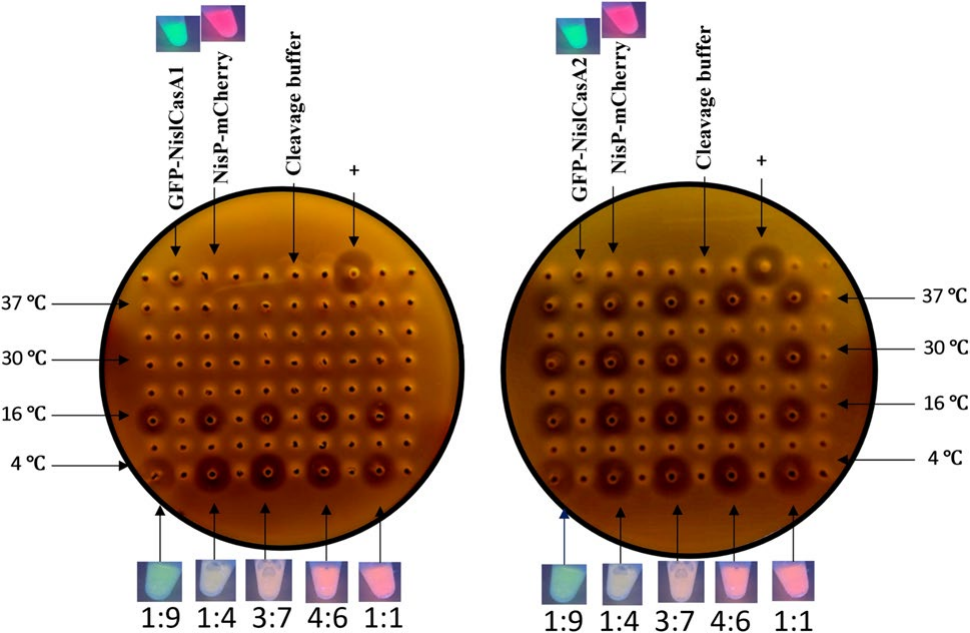
Cleavage optimization of GFP-CasA1 (left) and GFP-CasA2 (right) adding NisP-mCherry protease in different ratios; all conditions were then analyzed after 24 h of incubation at different temperatures. Cell-free pH adjusted (pH 7) supernatant from strain *Lact. plantarum* 423 (producer of plantaricin 423) was used as a positive control

Aiming to maximize the bacteriocin liberation from GFP protein, cleavage reactions were performed at a volume ratio of 1:9, 1:4, 3:7, 4:6, and 1:1 and incubated at 4, 16, 30, and 37 °C for 16 h. The highest antilisterial activity obtained after the cleavage of GFP-NislCasA1 and GFP-NislCasA2 was obtained after 16 h of incubation at 3:7 volume ratio and 4 °C (Fig. 4).

**Fig. 5:**
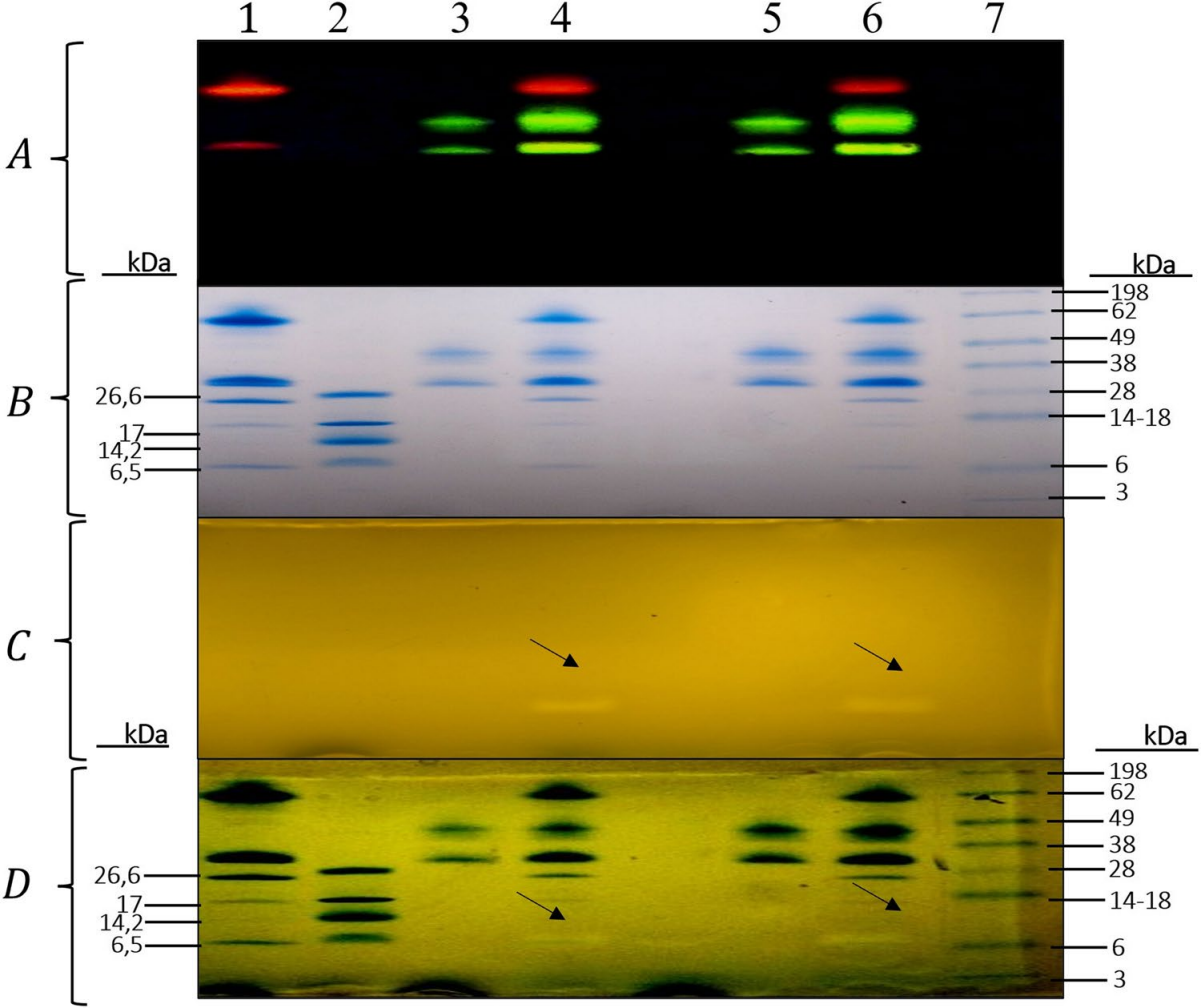
Tricine-SDS-PAGE analysis of casA1 and casA2 peptide liberation using NisP-mCherry. Lanes 1: NisP-mCherry, 2: Ultralowrange marker (M3456), 3: GFP-CasA1, 4: cleavage of GFP-CasA1 with NisP-mCherry, 5: GFP-CasA2, 6: cleavage of GFP-CasA2 with NisP-mCherry, 7: Seeblue plus ladder. While (A) presents the location of fluorescent NisP-mCherry and GFP-fusion constructs, no changes in GFP size were observed post-cleavage with NisP-mCherry (lanes 4 and 6). (B) Coomassie staining the gel presented in (A). (C) is the duplicate SDS-PAGE gel of (A) overlaid onto a BHI plate seeded with *L. monocytogenes* EDG-e where a zone of inhibition may be observed (black arrows). (D) Superimposition of (B) and (C) indicates that the inhibition zone corresponds to a band of approximately 5 kDa in size

The GFP-NislCasA1 and GFP-NislCasA2 fusion proteins cleaved with NisP-mCherry were analyzed by semi-native SDS-PAGE (Fig. 5). Although a size change could not be detected between uncleaved and cleaved GFP-NislCasA1 and GFP-NislCasA2, respectively, a peptide band could be detected confirming antilisterial activity for casA1 (5.1 kDa) and casA2 (5.2 kDa) (Fig. 5, black arrows) (Table 1).

**Table 1:** Plasmids, strains, and primers

	Description	Ref
Plasmid		
pRSF-GFP	Shuttle vector, Kan*	[36]
pRSF-hNisP	Shuttle vector, Kan*	[37]
pRSF-GFP-NislcasA1	6xHis-tag-GFP-Nisin leader peptide- CasA1 vector producer	This work
pRSF-GFP-NislcasA2	6xHis-tag-GFP-Nisin leader peptide- CasA2 vector producer	This work
pRSF-Nisp-mCherry8xHis	NisPmCherry-8xHis-tag peptide vector producer	This work
Strain		
*L. lactis* QU2	Nisin producer strain	[37]
*Lact. zeae* UD 2202	Strain under study with *casA1* gene	This work
*Lact. casei* UD 1001	Strain under study with *casA2* gene	This work
*E. coli* BL21 (DE3)	Expression host	
Primer		
GFPNisLeader_PstIF	GGAACTGCAGATGAGTACAAAAGA	This work
Rev_NisLeaderOri_casA1/2	CATAGTATTTGCGTGGTGATG	This work
FWNispL_casA1/2	CAGGTGCATCACCACGCAAATACTATGGTAATGGTGT	This work
PstCFbactFwd	GAACTGCAGAAATACTATGGTAATGGTG	This work
Hind_REV_casA1/2	GCAAAGCTTACTTGATGCCAGAATTC	This work
Fwd_mCherryNisP_Hind	GACAAGCTTTGGCAATCATCAAAGAATT	This work
Rev_mCherryNisP_Hind	GTCAAGCTTTATATAATTCATCCATACCAC	This work
pRSFMCS1_F	GGATCTCGACGCTCTCCCT	[37]
pRSFMCS1_R	GATTATGCGGCCGTGTACAA	[37]

^*^Kan, kanamycin resistance

Primer sequences represented in the 5’ to 3’ direction

### Mode of Action

The mature casA1 and casA2 liberation were evaluated by detecting antimicrobial activity against *L. monocytogenes* EDG-e before and after proteolytic cleavage.

**Fig. 6:**
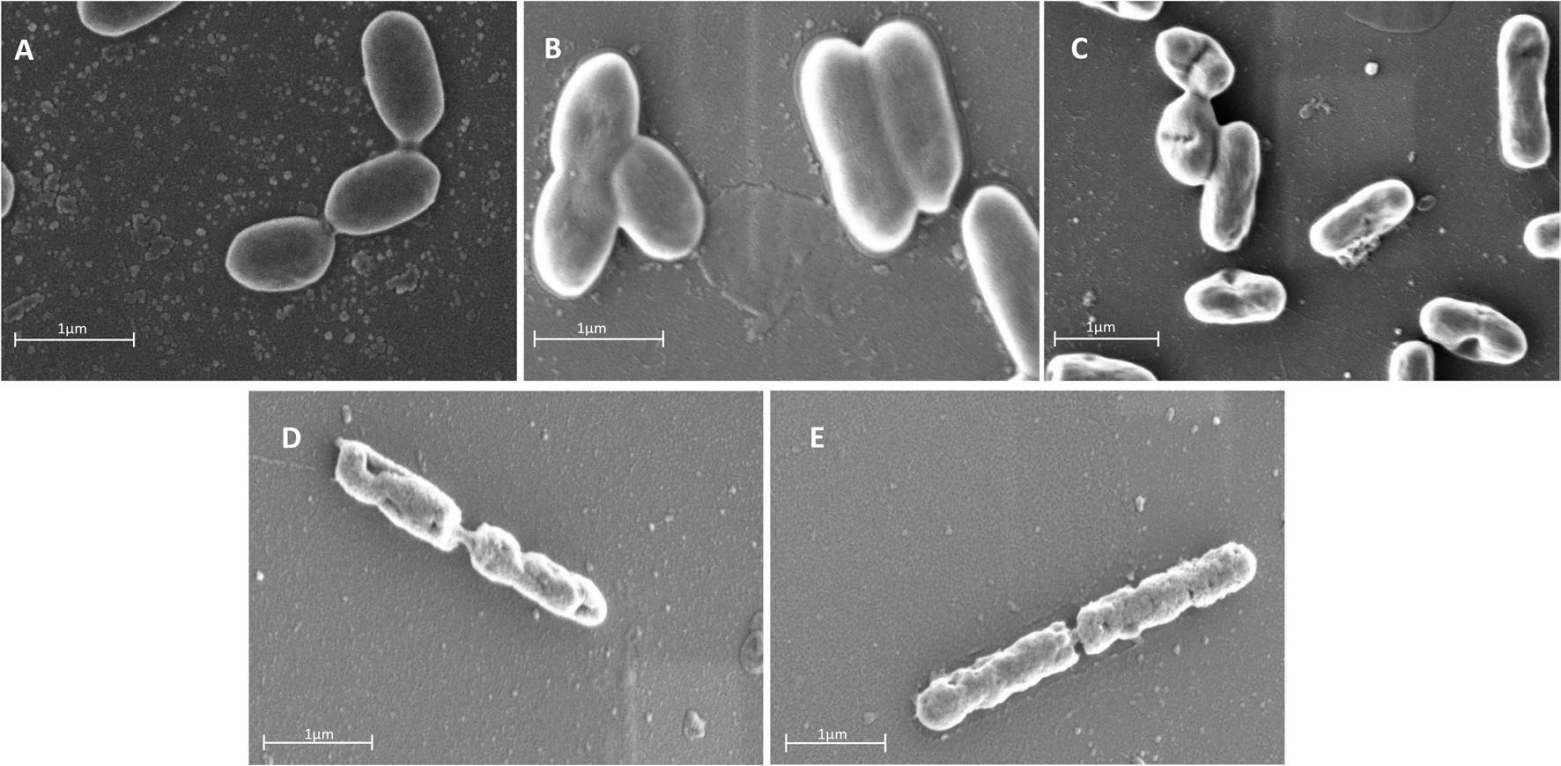
Mode of antimicrobial action for casA1 and casA2 against *L. monocytogenes* EDG-e cells observed under SEM. **A** Untreated cells, **B** treated with NisP-mCherry, **C** treated with GFP-Nisl-CasA1/CasA2, **D** treated with liberated CasA1, **E** treated with liberated casA2

Both liberated CasA1 and CasA2 peptides caused severe damage to the cell surface of *L. monocytogenes* EDG-e when compared to the untreated control or the NisP-mCherry protease (Fig. 6).

### Antimicrobial Spectrum and MIC

Active fractions of casA1 and casA2, obtained after HPLC separation and concentrated by lyophilization, revealed MIC values ranging from < 0.2 to 50 µg/mL (Table 2).

**Table 2:** Antibacterial spectra and MIC

Strain	CasA1 MIC (µg/mL)	CasA2 MIC (µg/mL)
*L. ivanovii* n29	< 0.2	< 0.2
*L. monocytogenes* ATCC 7646	0.35	0.35
*L. monocytogenes* RTE	6.25	6.25
*L. monocytogenes* EDG-e	12.5	12.5
*L. innocua* ATCC 33090	12.5	12.5
*Ent. faecium* HKLHS	25	25
*Ent. faecalis* S1	50	50
*Clos. difficile* ATCC 70057	50	50
*Lact. zeae* UD 2202	-	-
*Lact. casei* UD 1001	-	-

Both peptides exhibited potent activity against *Listeria* spp. and moderate activity against *Ent. faecium* HKLHS, *Ent. faecalis* S1, and* Clos. difficile* ATCC 70057. However, casA1 and casA2 were not active against the native producers *Lact. zeae* UD 2202 and *Lact. casei* UD 1001. Additional MIC results are shown in Fig. 4S.

## Discussion

The genes *casA1* and *casA2*, identified in two separate bacteriocin gene clusters (*Cas1* and *Cas2*), code for the production of two previously undescribed class IIa bacteriocins. The suffix “cin” was used in defining the name caseicin A1 (casA1) and caseicin A2 (casA2), as proposed by Montville and Kaiser [40]. Screening of cell-free supernatants produced by the wild-type producers, *Lact. zeae* UD 2202 and *Lact. casei* UD 1001, yielded no antimicrobial activity, despite sequence resemblance to other class II A bacteriocins. In silico mining of genome sequences provided valuable information on putative bacteriocin gene clusters. While metagenomic studies may be a viable source for discovering putative bacteriocin genes, the expression of these genes and the detection of antimicrobial active peptides can be challenging and require an in-depth understanding of gene regulation and peptide secretion. Expression of *casA1* and *casA2* in a heterologous host produced peptides with antimicrobial activity, demonstrating the strength of in silico mining and providing information required for the expression of putative bacteriocin genes [41, 42]. Information on the regulatory mechanisms used by bacteriocins has led to the development of plasmid-based expression systems and improved production levels [43, 44].

The His-tagged GFP-bacteriocin fusion protein heterologous expression system described by Vermeulen et al. [36, 45], yielded active bacteriocins (casA1 and casA2). The fluorescent protein (GFP) fused to the gene constructs made it possible to track the production of casA1 and casA2 during fermentation, extraction, and HPLC purification. According to Vermeulen et al. [36], the WELQut protease that is used to liberate peptides from the His-tagged GFP-bacteriocin fusion construct has an inefficient cleavage rate. In the present study, this was circumvented by using a heterologously produced NisP-mCherry protease. NisP is a specific membrane-anchored subtilisin-like serine peptidase (Pfam entry Peptidase S8) and plays a leading role in the last step of nisin maturation [46–48]. Mature class IIa bacteriocins, characterized by having one or more disulfide bridges and no lanthionine ring structures, prove that lanthionine is not essential for NisP activity, as also reported by Montalbán-López et al. [49].

Both peptides (casA1 and casA2) were heterologously expressed as GFP-fusion proteins (GFP-NislCasA1 and GFP-NislCasA2) in *E. coli* BL21 and were liberated from the GFP-fusion construct by using the NisP-mCherry protease. Cleavage reactions were performed in 200 mM NaCl and 50 mM Tris pH 7.5, without the requirement of additional co-factors. Differences observed in cleavage efficiency between CasA1 and CasA2 may be due to amino acid variations in position 19 (G and R). This change introduced an additional positive charged residue in CasA2, which increased the functional temperature range of NisP. The addition of the mCherry gene in the pRSF-NisP8xHis vector allowed for variations in temperature increases. Best results were obtained with freshly produced (heterologously expressed) NisP protease fused to the mCherry protein, suggesting that NisP may not be that stable in the fused construct. Further research is required to improve the stability of the NisP-mCherry construct.

Similar to other class IIa bacteriocins, casA1 and casA2 showed potent antilisterial activity and moderate activity against other closely related bacteria. Images obtained with SEM suggest that the mode of action is pore formation. Despite variations in amino acid compositions, no significant changes were detected in the specific activity of casA1 and casA2. This suggests that the additional net positive charge in casA2 has no selective advantage in bacterial defense. Based on the conserved “YGNGV” motif and their spectrum of antimicrobial activity, both peptides are classified as pediocin-like bacteriocins. This is supported by the cell membrane disruption observed [50, 51].

While the *Lact. zeae* UD 2202 and *Lact. casei* UD 1001 strains clearly show antimicrobial resistance to CasA1 and CasA2 peptides, antilisterial activity and, therefore, native expression of the CasA1 and CasA2 has not been detected from *Lact. zeae* UD 2202 and *Lact. casei* UD 1001, respectively. This might be due to a lack of niche-specific stimuli under laboratory culturing conditions. It is also possible that the wild-type strains only profit from having genes encoding immunity proteins, and hence protect themselves from other bacteriocin producers. The Cas2 operon appears to lack an intact ABC transporter and accessory protein, which suggests that *Lact. casei* UD 1001 lacks the ability to secrete an active CasA2 protein. This may further support the possibility that these operons are used for their immunity proteins only. To date, the mechanism by which immunity proteins induce resistance to the antibacterial activity of class IIa bacteriocins is not well described. It is currently unclear whether these immunity proteins act from an inter-, intra-, or extra-cellular position. If *Lact. zeae* UD 2202 and *Lact. casei* UD 1001 strains are genetically unable to secrete mature CasA1 and CasA2 peptides, this may indicate that the cognate immunity protein is also not secreted, however, both strains still actively were resistant to other external class IIa bacteriocins.

In the plethora of antimicrobial peptides, several bacteriocins have been discovered from *Lacticaseibacillus casei* spp. strains. These peptides include bacteriocin LiN333 [52], bacteriocin lactocin 705 [53, 54], bacteriocin caseicin 80 [55], bacteriocin LSEI_2163 [56], and bacteriocin caseicin TN-2 [57]. Interestingly, class IIa bacteriocin activity is yet to be described for strains of *Lact. casei* spp., although it is clear from this work that they do harbor the genetic potential to produce potent antibacterial class IIa peptides. Therefore, *Lact. casei* spp. might only produce class IIa bacteriocins under very niche-specific conditions, or they may harbor cryptic class IIa bacteriocin genes as a mechanism to gain a selective advantage through resistance without the metabolic burden of producing and secreting the active peptide.

## Conclusion

Metagenomic studies provide a vast body of information, which allows the comparison of data obtained by conventional microbiology techniques and simultaneously enhances the development of new technology. Extensive applications of predicational homology-based genomic tools are available to interpret genomic data.

Although CasA1 and CasA2 display classical class IIa bacteriocin activity, the expression of these peptides by the native producers *Lact. zeae* UD 2202 and *Lact. casei* UD 1001 was not observed. These operons may be cryptic due to a lack of niche-specific stimuli, or they may be maintained only for the benefit of the encoded immunity proteins. Future work should consider the potential regulatory mechanisms for these novel class IIa bacteriocins along with the effect resistant (and bacteriocin silent) bacteria have on population dynamics within the same niche. We propose to include casA1 and casA2 into the group of class IIa bacteriocins. Further research is being conducted on the regulation of the genes encoding casA1 and casA2.

## Supplementary Information

Below is the link to the electronic supplementary material.

Supplementary file1 (PPTX 6767 KB)

## Data Availability

No datasets were generated or analysed during the current study.
