# Supplementary material for: Expression of Caseicin from Lacticaseibacillus casei and Lacticaseibacillus zeae Provides Insight into Antilisterial Class IIa Bacteriocins
Source: Probiotics Antimicrob Proteins. 2024 Aug 13;17(6):3975–85. doi: 10.1007/s12602-024-10341-0 (PMC12634818; doi:10.1007/s12602-024-10341-0)
Supplement: Supplementary file 1 — Supplementary file1 (PPTX 6767 KB) [file 12602_2024_10341_MOESM1_ESM.pptx]

## Slide 1
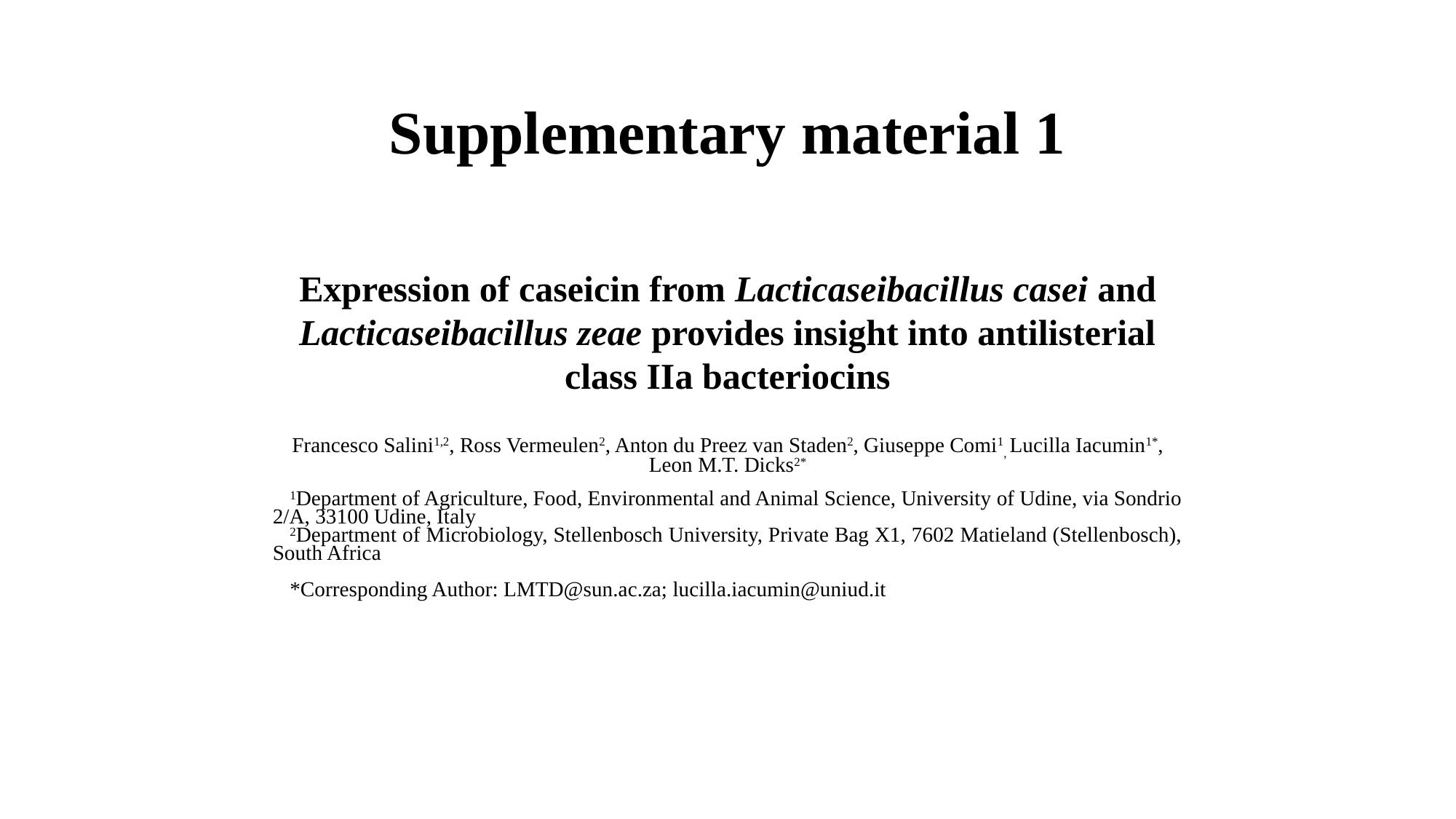

Supplementary material 1
Expression of caseicin from Lacticaseibacillus casei and Lacticaseibacillus zeae provides insight into antilisterial class IIa bacteriocins
Francesco Salini1,2, Ross Vermeulen2, Anton du Preez van Staden2, Giuseppe Comi1, Lucilla Iacumin1*, Leon M.T. Dicks2*
1Department of Agriculture, Food, Environmental and Animal Science, University of Udine, via Sondrio 2/A, 33100 Udine, Italy
2Department of Microbiology, Stellenbosch University, Private Bag X1, 7602 Matieland (Stellenbosch), South Africa
*Corresponding Author: LMTD@sun.ac.za; lucilla.iacumin@uniud.it

## Slide 2
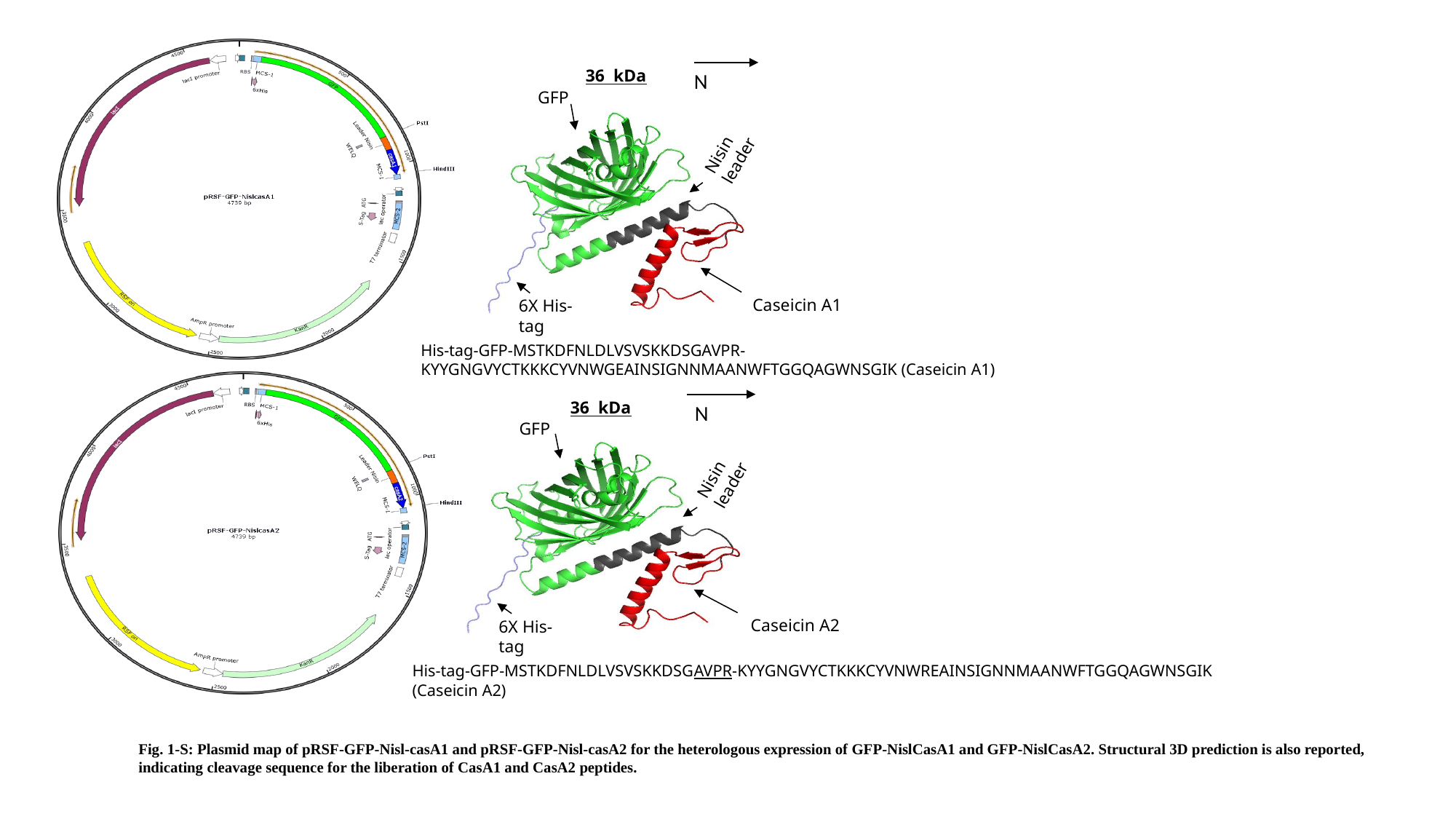

36 kDa
GFP
Nisin leader
6X His-tag
Caseicin A1
36 kDa
GFP
Nisin leader
6X His-tag
Caseicin A2
His-tag-GFP-MSTKDFNLDLVSVSKKDSGAVPR-KYYGNGVYCTKKKCYVNWGEAINSIGNNMAANWFTGGQAGWNSGIK (Caseicin A1)
His-tag-GFP-MSTKDFNLDLVSVSKKDSGAVPR-KYYGNGVYCTKKKCYVNWREAINSIGNNMAANWFTGGQAGWNSGIK (Caseicin A2)
Fig. 1-S: Plasmid map of pRSF-GFP-Nisl-casA1 and pRSF-GFP-Nisl-casA2 for the heterologous expression of GFP-NislCasA1 and GFP-NislCasA2. Structural 3D prediction is also reported, indicating cleavage sequence for the liberation of CasA1 and CasA2 peptides.

## Slide 3
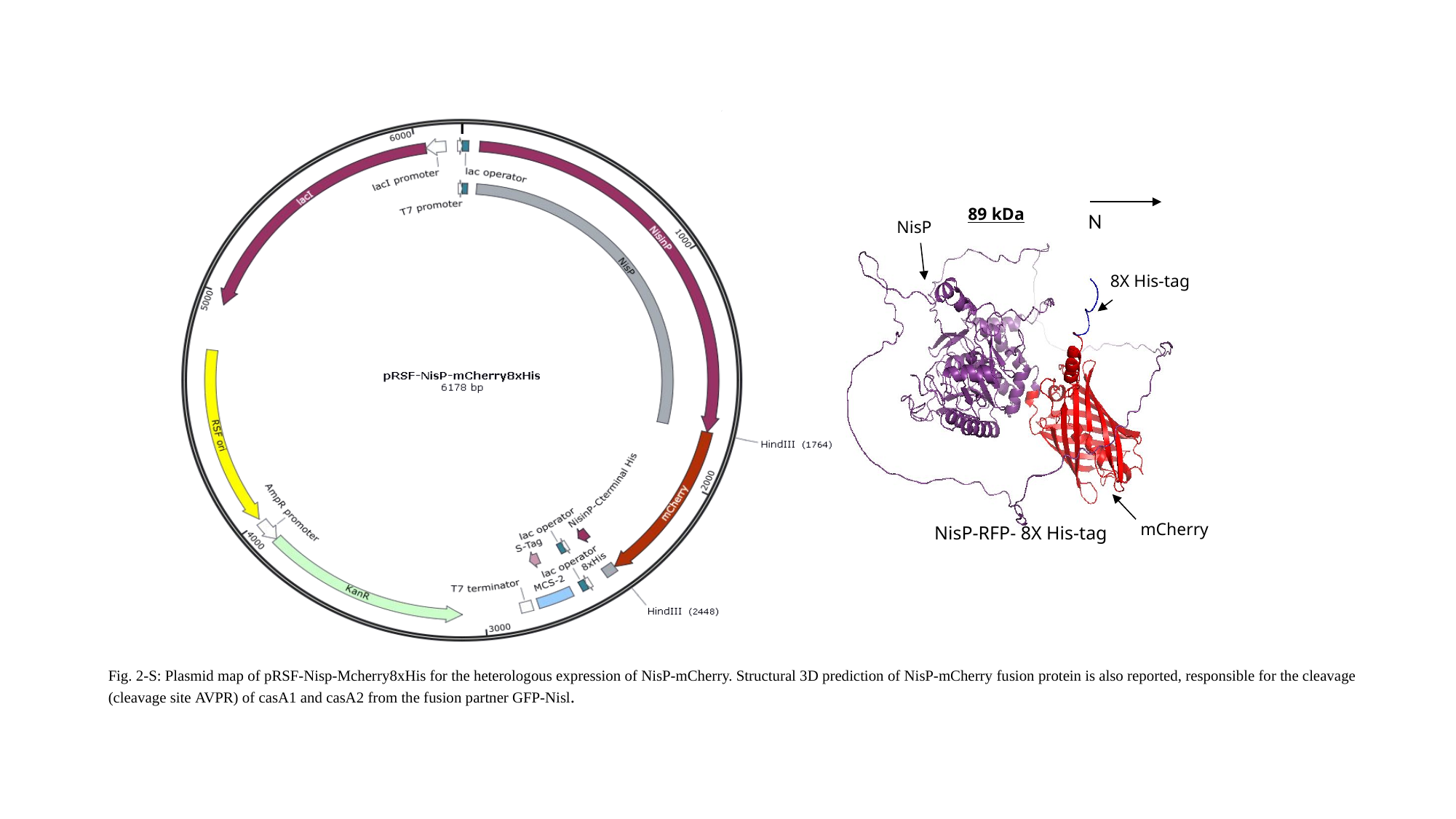

89 kDa
NisP
8X His-tag
mCherry
NisP-RFP- 8X His-tag
Fig. 2-S: Plasmid map of pRSF-Nisp-Mcherry8xHis for the heterologous expression of NisP-mCherry. Structural 3D prediction of NisP-mCherry fusion protein is also reported, responsible for the cleavage (cleavage site AVPR) of casA1 and casA2 from the fusion partner GFP-Nisl.

## Slide 4
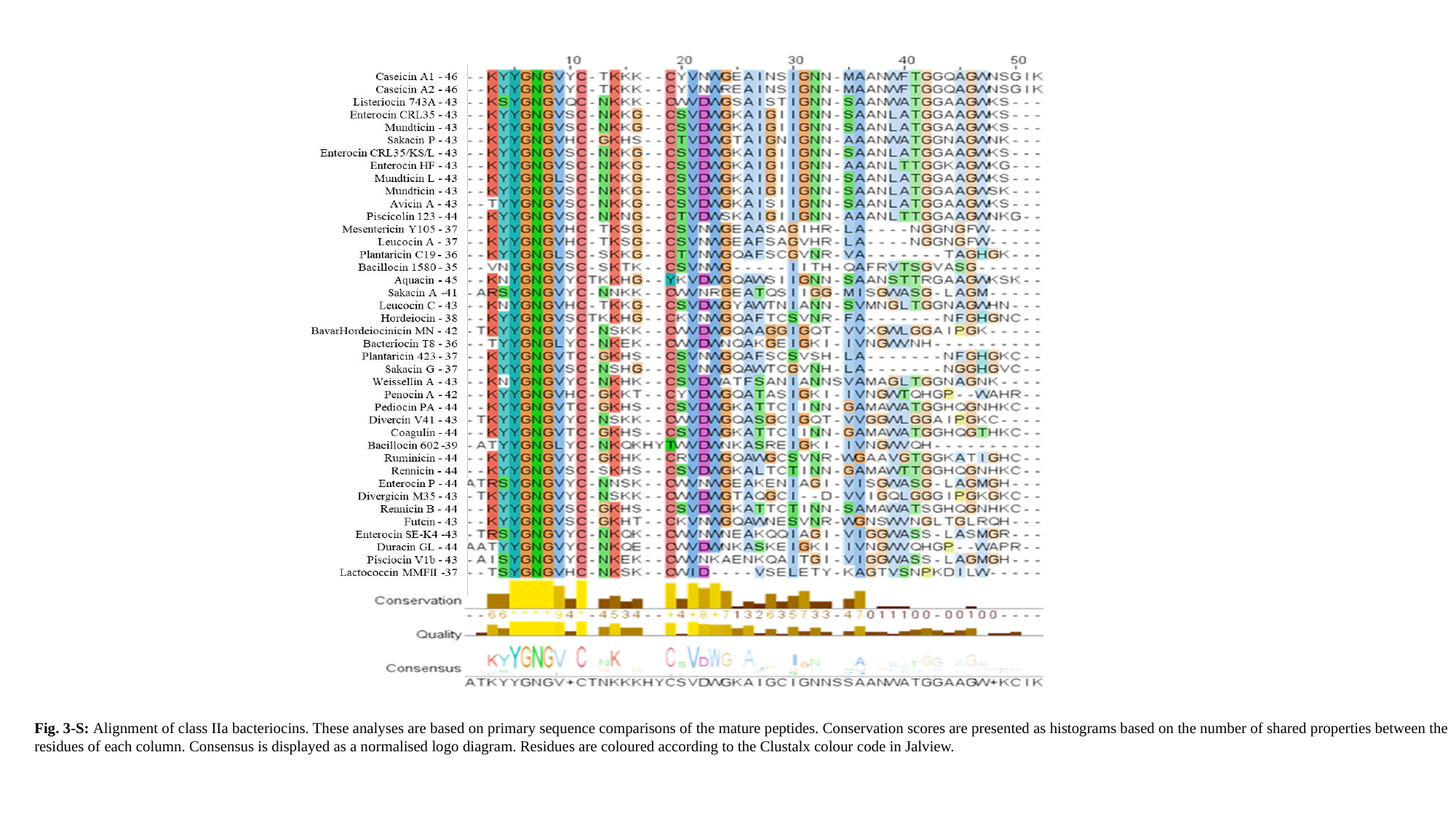

Fig. 3-S: Alignment of class IIa bacteriocins. These analyses are based on primary sequence comparisons of the mature peptides. Conservation scores are presented as histograms based on the number of shared properties between the residues of each column. Consensus is displayed as a normalised logo diagram. Residues are coloured according to the Clustalx colour code in Jalview.

## Slide 5
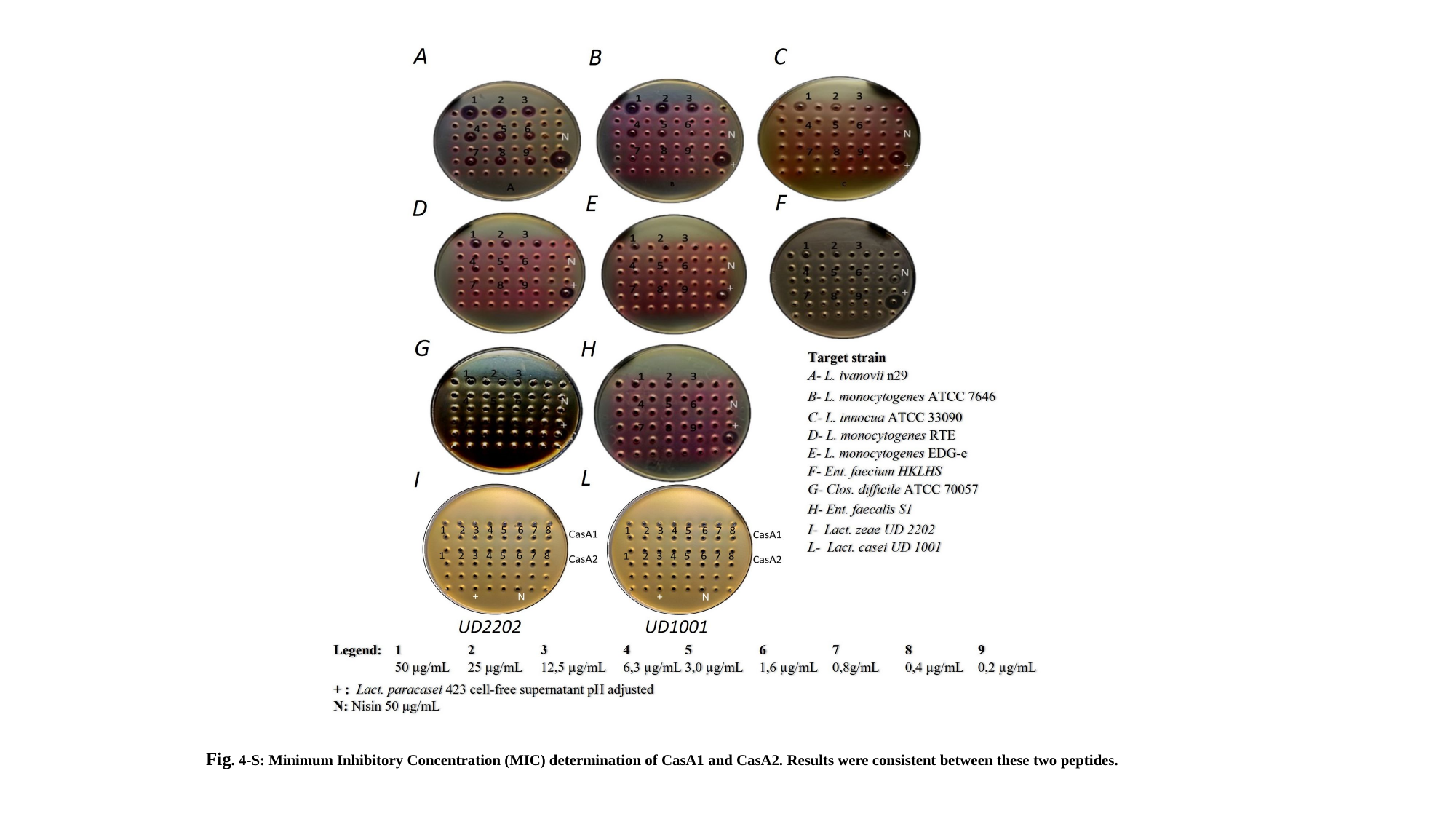

Fig. 4-S: Minimum Inhibitory Concentration (MIC) determination of CasA1 and CasA2. Results were consistent between these two peptides.
